# Trends in Comorbidity for Patients with Venous Thromboembolism in a General Hospital: 2018 to 2022

**DOI:** 10.3390/clinpract16060113

**Published:** 2026-06-15

**Authors:** Luisa Jiménez Reyes, José Javier Jareño Esteban, Lara Almudena Fernández Bermejo, Carlos Gutiérrez Ortega, Javier de Miguel-Díez

**Affiliations:** 1 Internal Medicine Department, Hospital Central de la Defensa “Gómez Ulla”, 28047 Madrid, Spain; 2 Respiratory Medicine Department, Hospital Central de la Defensa “Gómez Ulla”, 28047 Madrid, Spain; jjarest@mde.es; 3 Internal Medicine Department, Hospital de Santa Bárbara, 13500 Puertollano, Ciudad Real, Spain; lafb03@sescam.jccm.es; 4 Preventative Medicine Department, Hospital Central de la Defensa “Gómez Ulla”, 28047 Madrid, Spain; carlos.gutierrezo@uah.es; 5 Respiratory Medicine Department, Hospital Universitario Gregorio Marañón, 28047 Madrid, Spain; javier.miguel@salud.madrid.org

**Keywords:** pulmonary embolism, COVID-19, SARS-CoV-2, comorbidity, mortality

## Abstract

**Background/Objectives**: Recent trends show a rising incidence of venous thromboembolism (VTE) that does not correlate with increased mortality; however, population aging and the proliferation of comorbidities are fundamentally reshaping the VTE patient landscape. The aim of this study is to evaluate potential differences in clinical characteristics, comorbidities, and survival rates between patients diagnosed with pulmonary embolism (PE) during the pre-pandemic period (2018–2019) and those diagnosed during the pandemic era (2020–2022). Additionally, as a secondary objective, we analyze the clinical profiles, risk factors, and survival outcomes of patients with and without COVID-19 infection during the 2020–2022 period. **Methods**: A retrospective observational study was conducted to analyze survival and comorbidities in patients admitted for PE at the Hospital Central de la Defensa ‘Gómez Ulla’ between 2018 and 2022, comparing two periods (2018–2019 and 2020–2022). In addition, a sub-analysis was performed within the second period group comparing patients with and without COVID-19. **Results**: It was observed that the majority of patients in the first period were men, while in the second period, 55% were women. With regard to comorbidity and risk factors, thrombophilia and dementia were more prevalent in the first period, while asthma was more prevalent in the second period. No differences were found with regard to mortality. **Conclusions:** Significant differences were observed between the two periods of the study with regard to some comorbidities. Patients with COVID-19 showed a greater tendency toward immobilization and a higher prescription of thromboprophylaxis during hospitalization.

## 1. Introduction

In recent years, the incidence of venous thromboembolism (VTE) has increased significantly in Spain [1,2]. Concurrently, there has been a notable rise in the prevalence of comorbidities among these patients, including severe conditions such as malignancy and cardiovascular disease [3,4]. These factors contribute to a heightened risk of complications and, consequently, a poorer clinical prognosis.

While several studies indicate that the rising incidence of VTE has not been matched by a corresponding increase in mortality [5], a strong association between mortality rates and the burden of comorbidities remains evident [6,7,8]. Recently, the emergence of the COVID-19 pandemic, an aging population, immigration, and other factors may be leading to a change in the profile of patients with VTE.

The aim of this study is to evaluate potential differences in clinical characteristics, comorbidities, and survival rates between patients diagnosed with pulmonary embolism (PE) during the pre-pandemic period (2018–2019) and those diagnosed during the pandemic era (2020–2022). Additionally, as a secondary objective, we analyze the clinical profiles, risk factors, and survival outcomes of patients with and without COVID-19 infection during the 2020–2022 period.

## 2. Materials and Methods

A retrospective observational study was conducted to analyze survival and comorbidities in patients hospitalized for PE at the Hospital Central de la Defensa “Gómez Ulla” between 1 January 2018 and 31 December 2022. Diagnosis was confirmed via computed tomography pulmonary angiography (CTPA) and/or ventilation–perfusion lung scintigraphy, in accordance with the 2019 European Society of Cardiology (ESC) guidelines [9,10].

Data were extracted from inpatient clinical records, including sociodemographic variables, risk factors, and comorbidities. Thirty-day mortality risk was stratified using the Pulmonary Embolism Severity Index (PESI) and its simplified version (sPESI) [10]. Clinical symptoms presented during admission were also recorded.

A COVID-19 diagnosis was defined by compatible clinical symptoms confirmed by a positive nasopharyngeal RT-PCR test. Severe infection was classified according to the “Sociedad Española de Enfermedades Infecciosas y Microbiología Clínica” (SEIMC) criteria [11]. Immobilization was defined as bed rest or the inability to ambulate for more than four days. Postoperative PE was defined as occurring within two months of surgery, and long-distance travel was defined as a journey exceeding six hours within the three weeks preceding the thrombotic event.

The thrombophilias included were those with high thrombotic risk, both hereditary (homozygous *Factor V Leiden* mutation, homozygous prothrombin *G20210A* gene mutation, antithrombin deficiency, protein C deficiency, and protein S deficiency) and acquired (including antiphospholipid syndrome).

Quantitative variables are expressed as mean ± standard deviation (SD), while qualitative variables are presented as absolute frequencies and percentages. Associations between variables were assessed using the chi-square test. Precision was estimated using 95% confidence intervals (95% CIs). A multivariate binary logistic regression analysis was performed, including candidate variables with a bivariate *p*-value < 0.250. To address the “perfect separation issue” caused by the absence of COVID-19 in the first period, a sensitivity analysis was conducted. The final model was executed exclusively within the subsample of patients without COVID-19, expressing associations as odds ratios (ORs) with 95% confidence intervals (95% CIs).

Survival analysis was performed using the Kaplan–Meier method, with survival curve comparisons conducted via the log-rank test and Cox regression models. Statistical significance was set at *p* < 0.05. All analyses were performed using SPSS^®^ version 24.

The study was conducted in accordance with the ethical principles for medical research involving human subjects outlined in the Declaration of Helsinki. The protocol was approved by the Institutional Review Board (Medicines Research Ethics Committee of the General Health Inspectorate of Defense). All data were anonymized to ensure strict confidentiality, and access to protected health information was restricted in compliance with current data protection legislation.

## 3. Results

### 3.1. Study Population

A total of 321 patients were diagnosed with PE between 1 January 2018 and 31 December 2022. Of these, 35.2% were diagnosed during the 2018–2019 period, while 64.8% were diagnosed between 2020 and 2022.

**Figure 1:**
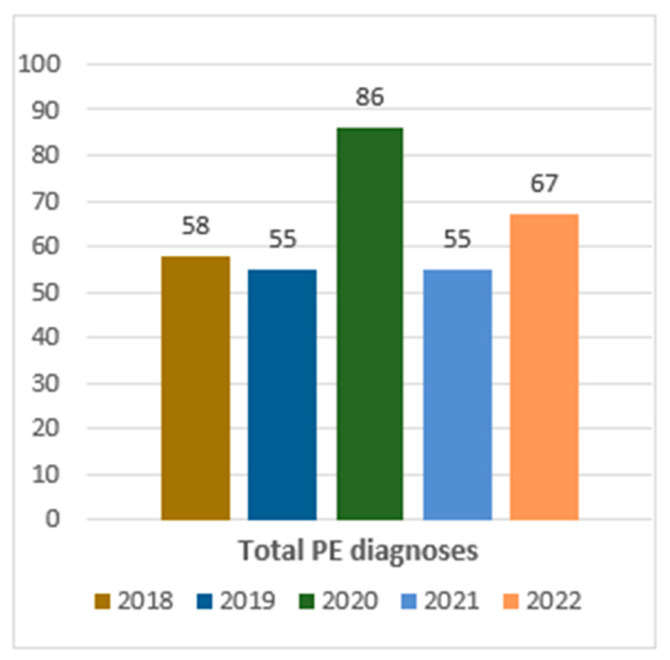
Bar chart illustrating the number of PE diagnoses from 2018 to 2022 at the Hospital Central de la Defensa “Gómez Ulla”.

The trend in diagnoses over this period is illustrated in Figure 1. Notably, a peak in the number of diagnoses occurred in 2020, coinciding with the COVID-19 pandemic, surpassing the figures recorded in the remaining years of the study.

Table 1 outlines the sociodemographic and epidemiological characteristics of the study patients. When comparing the two periods, significant differences were observed in the distribution by sex, with a predominance of females during 2020–2022 (55%), compared with a higher proportion of males in the 2018–2019 period.

Similarly, a history of thrombophilia and cancer, as well as treatments resulting from this diagnosis, and a history of recent surgery were more common in the first period, while immobilization was more common in the second.

Upon performing the multivariate analysis using logistic regression, we confirmed that thrombophilia was more frequent during the first period (OR: 2.62; 95% CI: 1.10–6.19; *p* = 0.028), as were a sedentary occupation (OR: 2.82; 95% CI: 1.24–6.43; *p* = 0.013) and having received radiotherapy (OR: 3.31; 95% CI: 1.43–7.67; *p* < 0.001).

With regard to clinical characteristics, as shown in Table 2, a higher incidence of chest pain, decompensated heart failure, hemodynamic instability, and dyspnea was observed during the first period of the study. The only one that remained significantly more frequent in the first period after multivariate analysis was chest pain (OR: 1.93; 95% CI: 1.1–3.38; *p* = 0.02).

Furthermore, the proportion of patients receiving thromboprophylaxis was higher during the second period; this is not confirmed by the multivariate analysis. No statistically significant differences were identified between the two periods for the remaining clinical variables. 

Risk stratification was performed using the PESI score, with significant differences observed between the two study periods. A higher proportion of patients was classified as PESI 1 during the 2020–2022 period, whereas a higher percentage was classified as PESI 2 during 2018–2019. When categorizing these groups as low (sPESI = 0) and high risk (sPESI ≥ 1), the prevalence of low-risk patients (sPESI 0) was 6.2% in the first period compared to 9.6% in the second.

### 3.2. Comorbidities and Survival

As for comorbidities, as shown in Table 3, statistically significant differences between periods were only identified for dementia, which was more prevalent in the first period, as well as for asthma, autoinflammatory and systemic diseases, and infections (including COVID-19 and other etiologies), all of which were more frequent in the second period.

Following a binary logistic regression model, asthma remained significantly associated with the second study period (OR = 8.28; 95% CI: 1.00–69.90; *p* = 0.049), while dementia remained significantly more frequent during the first period (OR = 2.64; 95% CI: 1.26–5.8; *p* = 0.001). 

**Figure 2:**
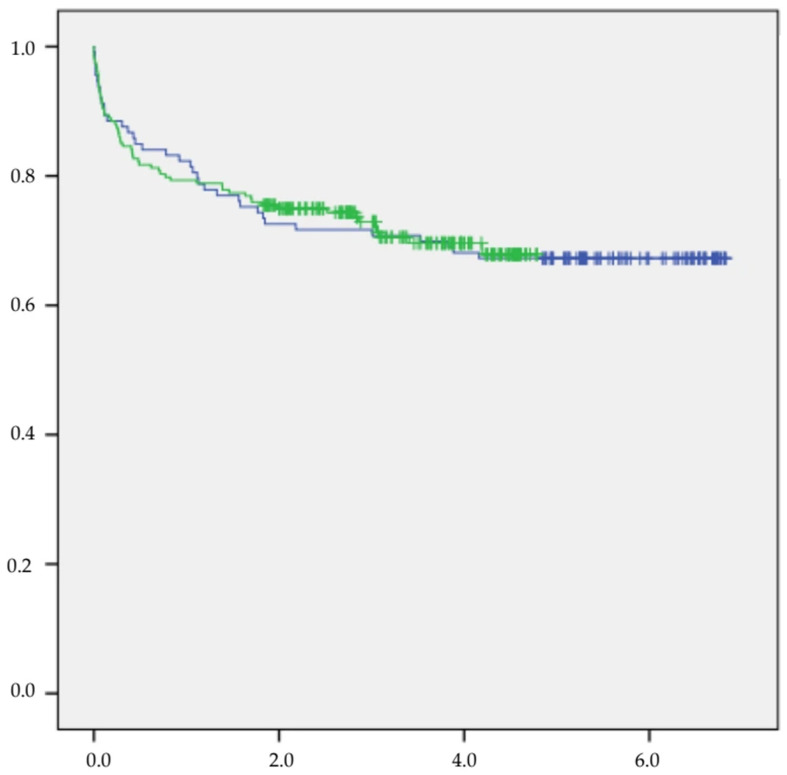
Kaplan–Meier curves for overall survival (years) by study period (2018–2019 (green) vs. 2020–2022 (blue)).

Regarding survival, no statistically significant differences were observed between the two periods, as illustrated in Figure 2.

### 3.3. Sociodemographic and Clinical Characteristics, Risk Factors, Comorbidities, and Survival During the 2020–2022 Period

As a secondary objective, we evaluated differences in sociodemographic and clinical characteristics, risk factors, comorbidities, and survival among patients diagnosed with PE during the 2020–2022 period, stratified by the presence or absence of COVID-19 infection.

As displayed in Table 4, this cohort included 207 patients, of whom 42 (20.3%) had COVID-19 infection, while 165 did not. The mean age was similar between the two groups. In the COVID-19 group, 52.4% were male, whereas the non-COVID-19 group showed a female predominance (57.6%). Furthermore, a higher proportion of patients under 70 years of age was observed in the COVID-19 group (50% vs. 33.9%, *p* = 0.05).

Regarding risk factors, the COVID-19 group exhibited a lower prevalence of cancer (4.8% vs. 34.5%, *p* < 0.001) and less frequent exposure to chemotherapy (2.4% vs. 16.4%, *p* = 0.01). Conversely, a higher prevalence of immobilization was observed in this group (76.2% vs. 18.2%, *p* < 0.001). No significant differences were found for the remaining variables.

As shown in Table 5, dyspnea was the most prevalent symptom in both groups, although it was significantly more frequent in the COVID-19 cohort (83.4% vs. 60.6%, *p* < 0.05). No significant differences were identified among the remaining symptoms.

Regarding thromboembolic complications, concomitant deep vein thrombosis (DVT) was significantly less frequent in the COVID-19 group (19% vs. 48.3%). Furthermore, a trend toward a lower rate of recurrent PE was observed in this group, although statistical significance was not reached.

Additionally, within our sample, a vast majority of patients with COVID-19 received thromboprophylaxis (73.8%) compared to only 2.4% of patients without a COVID-19 infection (*p* = 0.001).

Concerning hemorrhagic events, the incidence was low in both groups, including major gastrointestinal, urological, and intracranial bleeding, as well as hematomas, with no significant differences observed.

Concerning risk stratification using the PESI score, a trend toward a higher percentage of high-risk patients was noted in the COVID-19 group, although these differences did not reach statistical significance.

As can be seen in Table 6, the only comorbidity that showed significant differences between groups was non-COVID-19 infection, which was more prevalent in the COVID-19 cohort. 

**Figure 3:**
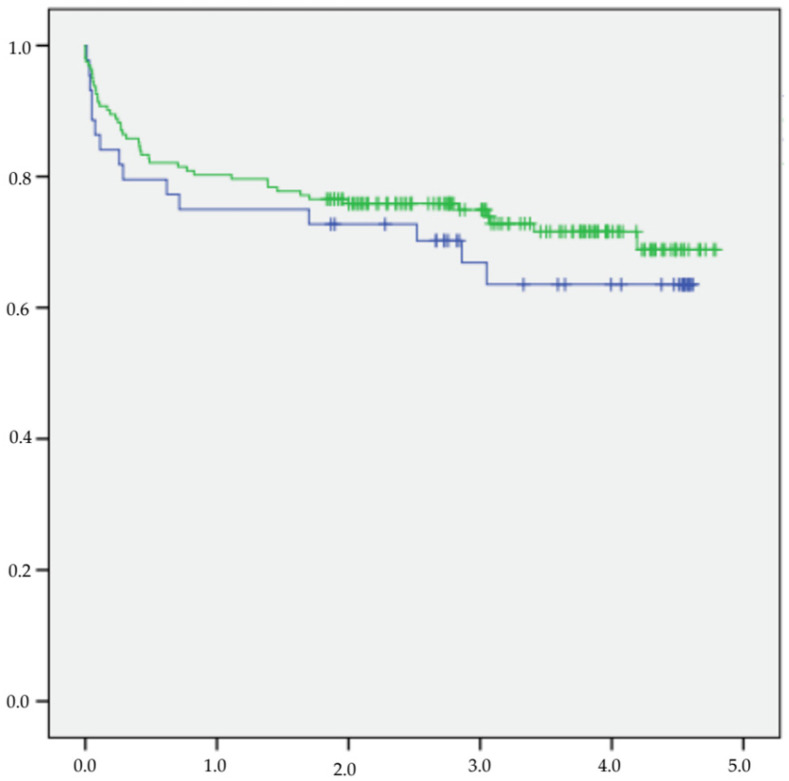
Kaplan–Meier survival curves for patients with (blue) and without (green) COVID-19 infection.

Regarding survival, no significant differences were found between the two groups, neither in the first 30 days nor in the long term (*p* = 0.3), as can be seen in Figure 3. 

## 4. Discussion

COVID-19 infection is associated with an increased risk of VTE, particularly in hospitalized patients or those with severe disease [12,13]. Multiple mechanisms promote the hypercoagulable state described in these patients, including endothelial dysfunction, platelet activation, and alterations in coagulation and fibrinolysis [14,15].

In this single-center study, which analyzed 321 patients diagnosed with acute PE between 2018 and 2022, patients were stratified into two groups: 2018–2019 and 2020–2022. Both study groups were homogeneous, with a similar mean age; however, differences were found in sex distribution, with a higher proportion of males in the first period and females in the second.

Numerous studies have shown that patients with VTE in the context of COVID-19 infection exhibit a different profile compared to those without the infection [16,17]. However, these differences were not evident in our analysis, which may be explained by the inclusion of non-infected patients in the 2020–2022 group, representing nearly 80% of that cohort.

Regarding risk factors and sociodemographic characteristics, thrombophilia, radiotherapy history, sedentary occupation, recent surgery, and thromboprophylaxis use were initially more frequent in the first group. After adjusting for confounding through a multivariate analysis that excluded COVID-19 patients, only thrombophilia, sedentary lifestyle, and radiotherapy treatment remained significantly associated with the first period. Immobilization and thromboprophylaxis showed no significant differences in this final model. This suggests COVID-19 acted as a confounder, given that infected patients underwent microbiological isolation and room confinement, leading to restricted ambulation that mandated thromboprophylaxis. We did not identify other potential confounders or treatments that could influence the clinical course of PE.

Regarding comorbidities, the bivariate analysis showed that asthma, systemic autoimmune diseases, and active infection (due to causes other than SARS-CoV-2) were more prevalent in the second period, whereas dementia was more frequent in the first. In the multivariate analysis, only asthma remained significantly more frequent in the second period, whereas dementia remained more frequent in the first. Notably, the confidence interval for asthma is strikingly wide; this low precision may be due to the small sample size. Likewise, SARS-CoV-2 infection may act as a confounder for active infection, given that patients with COVID-19 pneumonia frequently develop secondary infections, particularly respiratory ones.

No statistically significant differences were observed for the remaining variables studied.

This finding contrasts with other studies, such as that by Alonso-Beato et al. [16], who analyzed VTE episodes in patients with and without COVID-19. They observed differences between both groups, particularly regarding risk factors and comorbidities; specifically, smoking, ischemic heart disease, and, consistent with our study, immobilization were more frequent in the COVID-19 group. Nevertheless, unlike our findings, they reported no differences in cancer prevalence between the two groups. Similarly, a meta-analysis published in 2021 reported that traditional risk factors typically associated with VTE are often absent in VTE cases associated with COVID-19 infection [17].

Regarding clinical presentation, patients with COVID-19 infection exhibited a higher frequency of chest pain. A meta-analysis by Suh et al. [18] reported that the rate of DVT in patients with COVID-19 could reach 50%. However, the DVT rate in our study was considerably lower, which might be attributed to underdiagnosis during the pandemic period.

Although some studies report higher short-term mortality associated with SARS-CoV-2 infection, no significant differences were observed in our sample for either short- or long-term mortality. For instance, Alonso-Beato et al. observed that mortality at 30 days and one year was higher in the COVID-19 group [16]. Similarly, a European cohort study involving a large population found that mortality was higher among patients aged 65 years or older with COVID-19 infection in both inpatient and outpatient settings [13].

Furthermore, no increased risk of acute bleeding was observed in patients with COVID-19 infections, contrary to findings reported in other studies [19].

Our study has several limitations. First, its observational and retrospective design precludes the establishment of causal relationships, allowing only for the identification of associations. Second, the small sample size, particularly within the COVID-19 subgroup, limits the statistical power of our findings.

## 5. Conclusions

In our study population, an increase in the number of PE diagnoses was observed during 2020, coinciding with the onset of the COVID-19 pandemic. However, in subsequent years, diagnostic rates returned to levels similar to those recorded in 2018 and 2019.

When comparing the 2018–2019 and 2020–2022 periods, significant differences were identified in certain patient characteristics, particularly in specific comorbidities. Specifically, conditions such as asthma were more frequent during the second analyzed period, while thrombophilia and dementia were more prevalent in the first period.

Additionally, patients with COVID-19 infection exhibited a greater tendency toward immobilization during hospitalization and received thromboprophylaxis more frequently. Conversely, our study found no statistically significant differences between the two periods in terms of survival, PE recurrence, or bleeding risk, findings that contrast with certain results reported in previous literature. Finally, prognostic stratification using the PESI score allowed for the identification of specific differences in the distribution of low- and high-risk PE patients between the two analyzed periods; however, these differences did not reach statistical significance.

## Figures and Tables

**Table 1:** Sociodemographic and epidemiological characteristics and risk factors for the periods 2018–2019 and 2020–2022.

		2018–2019 n (%) 113 (35.2)	2020–2022 n (%) 208 (64.8)	Total n = 321	*p*
Age (years), mean (SD)		73.8 (±13.4)	72.3 (±15.8)	76 (±15.8)	0.3
Sex, n (%)					
	Male	65 (57.5)	93 (44.7)	158 (49.2)	0.02 *
	Female	48 (42.5)	115 (55.3)	163 (50.8)	
Age < 70 years		32 (28.3)	77 (37)	109 (34)	0.1 *
Smoking status, n (%)					
	Never smoker	71 (63.4)	139 (67.1)	210 (65.8)	0.4 *
	Former smoker	28 (25)	40 (19.3)	68 (21.3)	
	Current smoker	13 (12.9)	28 (13.5)	41 (12.9)	
Oral contraceptive use, n (%)		3 (2.7)	5 (2.4)	8 (2.5)	1 **
Sedentary occupation, n (%)		20 (17.8)	21 (10.1)	41 (12.8)	0.05 *
Cancer history, n (%)		39 (34.5)	59 (28.5)	98 (30.6)	0.3 *
	In remission	11 (32.4)	18 (32.1)	29 (32.2)	0.4 *
	Localized	9 (26.5)	9 (16.1)	18 (20)	
	Advanced	14 (41.2)	29 (51.8)	43 (47.8)	
Hormone therapy, n (%)		7 (6.2)	8 (3.9)	15 (4.7)	0.3 *
Tumor surgery, n (%)		17 (15)	27 (13)	44 (13.8)	0.6 *
Chemotherapy, n (%)		21 (18.6)	28 (13.5)	49 (15.3)	0.2 *
Radiotherapy, n (%)		18 (15.9)	12 (5.8)	30 (9.4)	0.003 *
Immunotherapy, n (%)		3 (1.7)	3 (1.4)	6 (1.9)	0.6 **
Thrombophilia, n (%)		16 (14.5)	12 (5.8)	28 (8.8)	0.009 *
Recent surgery, n (%)		20 (17.9)	18 (8.6)	38 (11.8)	0.02 **
	Major orthopedic surgery	2 (1.8)	9 (4.3)	11 (3.4)	
	Abdominal surgery	13 (11.6)	8 (3.8)	21 (6.6)	
	Thoracic surgery	2 (1.8)	0	2 (0.6)	
	Intracranial surgery	0	1 (0.5)	1 (0.3)	
	Other	3 (2.7)	0	3 (0.9)	
Long-distance travel, n (%)		3 (2.7)	2 (1)	5 (1.6)	0.3 **
Family history of PE, n (%)		1 (4.4)	9 (4.3)	14 (4.4)	1 **
Previous VTE, n (%)		22 (19.5)	31 (15)	53 (16.6)	0.3 *
Prolonged immobility, n (%)		17 (15.2)	62 (30)	79 (24.8)	0.004 *

* Pearson’s chi-square test, ** Fisher’s exact test.

**Table 2:** Clinical characteristics for the periods 2018–2019 and 2020–2022.

		2018–2019 n (%) 113 (35.2)	2020–2022 n (%) 208 (64.8)	Total 321	*p*
Syncope, n (%)		19 (16.8)	24 (11.6)	43 (13.4)	0.1 *
Chest pain, n (%)		43 (38.1)	52 (25.1)	95 (29.7)	0.01 *
Hemoptysis, n (%)		3 (2.7)	2 (1)	5 (1.6)	0.2 **
Acute heart failure, n (%)		14 (12.4)	10 (4.9)	24 (7.5)	0.01 *
Hemodynamic instability, n (%)		17 (15)	18 (8.7)	35 (10.9)	0.07 *
Dyspnea (mMRC ≥ 3), n (%)		83 (73.2)	135 (65.2)	218 (67.9)	0.2 *
Incidental PE, n (%)		15 (13.4)	15 (7.2)	30 (9.4)	0.07 *
Concomitant DVT, n (%)		55 (49.1)	87 (42)	142 (44.5)	0.2 *
Recurrent PE, n (%)		14 (12.4)	19 (9.2)	33 (10.3)	0.4 *
Thromboprophylaxis, n (%)		3 (2.7)	35 (16.9)	38 (11.9)	<0.001 **
Bleeding, n (%)					
	Gastrointestinal	4 (3.5)	4 (1.9)	8 (2.5)	0.1 **
	Urological	1 (2.7)	1 (0.5)	4 (1.3)	
	Intracranial	4 (3.5)	3 (1.4)	7 (2.2)	
	Hematoma	7 (6.2)	4 (1.9)	11 (3.4)	
PESI, n (%)					
	1	5 (4.5)	21 (10.1)	25 (8.1)	0.3 *
	2	15 (13.4)	33 (15.9)	48 (15)	
	3	24 (21.4)	48 (23.1)	72 (22.5)	
	4	26 (23.2)	37 (17.8)	63 (19.7)	
	5	42 (37.5)	68 (32.7)	110 (34.4)	
sPESI					
	0	7 (6.2)	20 (9.6)	24 (8.4)	0.003 **
	1	20 (17.7)	68 (32.7)	88 (27.4)	
	2	52 (46)	58 (27.9)	110 (34.3)	
	3	28 (23)	40 (19.2)	66 (20.6)	
	4	6 (5.3)	21 (10.1)	27 (8.4)	
	5	2 (1.8)	1 (0.5)	3 (0.9)	

* Pearson’s chi-square test, ** Fisher’s exact test.

**Table 3:** Comorbidities in the periods 2018–2019 and 2020–2022.

		2018–2019 n (%) 113 (35.2)	2020–2022 n (%) 208 (64.8)	Total 321	*p*
COVID-19 pneumonia, n (%)		0	42 (20.3)	42 (13.1)	<0.001 **
COPD, n (%)		15 (13.3)	29 (14)	44 (13.8)	0.8 *
Asthma, n (%)		1 (0.9)	15 (7.2)	16 (5)	0.01 **
Pulmonary fibrosis, n (%)		2 (1.8)	4 (1.9)	6 (1.9)	0.9 **
OSA, n (%)		15 (13.3)	31 (15)	46 (14.4)	0.6 *
Gastroesophageal Reflux Disease, n (%)		9 (8)	13 (22)	22 (6.9)	0.5 *
Peripheral artery disease, n (%)		4 (3.9)	5 (2.4)	9 (2.8)	0.5 **
Pulmonary hypertension, n (%)		18 (15.9)	31 (15)	49 (15.3)	0.8 *
Dyslipidemia, n (%)		46 (40.7)	88 (42.5)	134 (41.9)	0.7 *
Hypertension, n (%)		72 (63.7)	127 (61.4)	199 (62.2)	0.6 *
DM, n (%)		27 (23.9)	50 (24.2)	77 (24.1)	0.9 *
DM with TOD, n (%)		2 (1.8)	4 (1.9)	6 (1.9)	0.9 **
Hemiplegia, n (%)		3 (2.7)	3 (1.4)	6 (1.9)	0.4 **
Ischemic heart disease, n (%)		10 (8.8)	15 (7.2)	25 (7.8)	0.6 *
SAD, n (%)		0	8 (3.9)	8 (2.5)	0.03 **
Peptic ulcer disease, n (%)		2 (1.8)	6 (2.9)	8 (2.5)	0.5 **
Dementia, n (%)		21 (18.6)	22 (10.6)	43 (13.4)	0.04 *
Stroke, n (%)		6 (5.3)	12 85.8)	18 (5.6)	0.8 *
Moderate-to-severe CKD, n (%)		7 (6.2)	18 (8.7)	25 (7.8)	0.4 *
Mild liver disease, n (%)		5 (4.4)	6 (2.9)	11 (3.4)	0.4 *
Moderate-to-severe liver disease, n (%)		2 (1.8)	6 (2.9)	8 (2.5)	0.5 **
Obesity, n (%)		23 (20.4)	33 (15.9)	56 (17.5)	0.3 *
Severe mental illness		5 (4.4)	18 (8.7)	23 (7.2)	0.1 *
	Schizophrenia, n (%)	2 (1.8)	1 (0.5)	3 (0.9)	0.8 **
	Bipolar disorder, n (%)	2 (1.8)	4 (1.9)	6 (1.9)	
	Major depression, n (%)	1 (0.9)	9 (4.3)	10 (3.1)	
	Intellectual disability, n (%)	0	4 (1.9)	4 (1.3)	
Active infection, n (%)		13 (11.5)	48 (23.2)	61 (19.1)	0.01 *

* Pearson’s chi-square test, ** Fisher’s exact test.

**Table 4:** Epidemiological and sociodemographic characteristics of patients with and without COVID-19 infection, 2020–2022.

		COVID-19 n (%) 42 (20.3)	No COVID-19 n (%) 165 (79.7)	*p*
Age (years), mean (SD)		71.3 (±16.1)	72.4 (±15.7)	0.3
Sex, n (%)				
	Male	22 (52.4)	70 (42.4)	0.2 *
	Female	20 (47.6)	95 (57.6)	
Age < 70 years		21 (50)	56 (33.9)	0.05 *
Smoking status, n (%)				
	Never smoker	32 (76.2)	107 (64.8)	0.3 **
	Former smoker	6 (14.3)	34 (20.6)	
	Current smoker	3 (9.5)	24 (14.5)	
Oral contraceptive use, n (%)		0	5 (3)	0.3 **
Sedentary occupation, n (%)		5 (11.9)	15 (9.1)	0.5 *
Cancer history, n (%)		2 (4.8)	57 (34.5)	<0.001 **
	In remission	1 (50)	17 (31.5)	0.7 **
	Localized	0	9 (16.1)	
	Advanced	1 (50)	28 (51.9)	
Hormone therapy, n (%)		0	8 (4.8)	0.1 **
Tumor surgery, n (%)		2 (4.8)	25 (15.2)	0.07 **
Chemotherapy, n (%)		1 (2.4)	27 (16.4)	0.01 **
Radiotherapy, n (%)		0	12 (7.3)	0.07 **
Immunotherapy, n (%)		0	3 (1.8)	0.5 **
Thrombophilia, n (%)		1 (2.4)	11 (6.7)	0.4 **
Recent surgery, n (%)		0	18 (10.9)	0.1 **
	Major orthopedic surgery	0	9 (5.5)	0.1 **
	Abdominal surgery	0	8 (3.9)	
	Thoracic surgery	0	0	
	Intracranial surgery	0	1 (0.5)	
	Other	0	0	
Long-distance travel, n (%)		0	2 (1.2)	0.9 **
Family history of PE, n (%)		1 (2.4)	8 (4.8)	0.6 **
Previous VTE, n (%)		3 (7.1)	28 (17.1)	0.1 **
Prolonged immobility, n (%)		32 (76.2)	30 (18.2)	<0.001 *

* Pearson’s chi-square test, ** Fisher’s exact test.

**Table 5:** Clinical characteristics of patients with and without COVID-19 infection, 2020–2022.

	COVID-19 n (%) 42 (20.3)	No COVID-19 n (%) 165 (79.7)	*p*
Syncope, n (%)	2 (4.8)	22 (13.3)	0.1 **
Chest pain, n (%)	7 (16.7)	45 (27.3)	1 *
Hemoptysis, n (%)	0	2 (1.2)	0.6 **
Acute heart failure, n (%)	1 (2.4)	9 (5.5)	0.6 **
Hemodynamic instability, n (%)	5 (11.9)	13 (7.9)	0.3 *
Dyspnea (mMRC ≥ 3), n (%)	35 (83.4)	100 (60.6)	0.004 *
Incidental PE, n (%)	2 (4.8)	13 (7.9)	0.7 **
Concomitant DVT, n (%)	8 (19)	79 (48.3)	0.001 *
Recurrent PE, n (%)	1 (2.4)	18 (10.9)	0.1 **
Thromboprophylaxis, n (%)	31 (73.8)	4 (2.4)	0.001 **
Bleeding, n (%)			
Gastrointestinal	1 (2.4)	3 (1.8)	0.6 **
Urological	0	1 (0.6)	
Intracranial	1 (2.4)	2 (1.2)	
Hematoma	2 (2.8)	2 (1.2)	
sPESI			
Low risk (sPESI = 0)	1 (2.4)	19 (11.5)	0.07 **
High risk (sPESI ≥ 1)	41 (97.6)	146 (88.5)	

* Pearson’s chi-square test, ** Fisher’s exact test.

**Table 6:** Comorbidities of patients with and without COVID-19 infection, 2020–2022.

		COVID-19 n (%) 42 (20.3)	No COVID-19 n (%) 165 (79.7)	*p*
COVID-19 pneumonia, n (%)		6 (14.3)	23 (13.9)	0.9 *
COPD, n (%)		3 (7.1)	12 (7.3)	0.9 **
Asthma, n (%)		2 (4.8)	2 (1.2)	0.1 **
Pulmonary fibrosis, n (%)		1 (2.4)	12 (7.3)	0.4 **
OSA, n (%)		0	5 (3)	0.2 **
Gastroesophageal Reflux Disease, n (%)		3 (7.1)	28 (17)	0.1 **
Peripheral artery disease, n (%)		20 (47.6)	68 (41.2)	0.4 *
Pulmonary hypertension, n (%)		23 (54.8)	104 (63)	0.3 *
Dyslipidemia, n (%)		14 (33.3)	36 (21.8)	0.1 *
Hypertension, n (%)		0	4 (2.4)	0.5 **
DM, n (%)		0	3 (1.8)	0.9 **
DM with TOD, n (%)		5 (11.9)	10 (6.1)	0.1*
Hemiplegia, n (%)		6 (14.3)	19 (11.5)	0.6 *
Ischemic heart disease, n (%)		2 (4.8)	6 (3.6)	0.6 **
SAD, n (%)		1 (2.4)	5 (3)	0.6 **
Peptic ulcer disease, n (%)		3 (7.1)	19 (11.5)	0.4 **
Dementia, n (%)		0	12 (7.3)	0.1 **
Stroke, n (%)		3 (7.1)	15 (9.1)	0.4 **
Moderate-to-severe CKD, n (%)		1 (2.4)	5 (3)	0.6 **
Mild liver disease, n (%)		0	6 (3.6)	0.3 **
Moderate-to-severe liver disease, n (%)		7 (16.7)	26 (15.8)	0.8 *
Obesity, n (%)		3 (7.1)	15 (9.1)	0.6 **
	Schizophrenia, n (%)	0	1 (0.6)	0.6 **
	Bipolar disorder, n (%)	0	4 (2.4)	
	Major depression, n (%)	2 (4.8)	7 (4.2)	
	Intellectual disability, n (%)	1 (2.4)	3 (1.8)	
Active infection, n (%)		30 (71.4)	18 (10.9)	<0.001 **

* Pearson’s chi-square test, ** Fisher’s exact test.

## Data Availability

The original contributions presented in this study are included in the article. Further inquiries can be directed to the corresponding author.

## Abbreviations

The following abbreviations are used in this manuscript:

VTE	venous thromboembolism
PE	pulmonary embolism
DVT	deep vein thrombosis
CTPA	computed tomography pulmonary angiography
ESC	European Society of Cardiology
PESI	Pulmonary Embolism Severity Index
sPESI	simplified Pulmonary Embolism Severity Index
SEIMC	Sociedad Española de Enfermedades Infecciosas y Microbiología Clínica (Spanish Society of Infectious Diseases and Clinical Microbiology)
RT-PCR	reverse transcription polymerase chain reaction
SD	standard derivation
CI	confidence interval
COPD	chronic obstructive pulmonary disease
OSA	obstructive sleep apnea
DM	diabetes mellitus
TOD	target organ damage
SAD	systemic autoimmune disease
CKD	chronic kidney disease
mMRC	modified Medical Research Council
